# Engineering Organoids for *in vitro* Modeling of Phenylketonuria

**DOI:** 10.3389/fnmol.2021.787242

**Published:** 2022-01-10

**Authors:** Alice C. Borges, Kerensa Broersen, Paula Leandro, Tiago G. Fernandes

**Affiliations:** ^1^Department of Bioengineering and iBB – Institute for Bioengineering and Biosciences, Instituto Superior Técnico, Universidade de Lisboa, Lisbon, Portugal; ^2^Associate Laboratory i4HB – Institute for Health and Bioeconomy, Instituto Superior Técnico, Universidade de Lisboa, Lisbon, Portugal; ^3^Department of Applied Stem Cell Technologies, Faculty of Science and Technology, Technical Medical Centre, University of Twente, Enschede, Netherlands; ^4^Faculty of Pharmacy, iMed.ULisboa - Research Institute for Medicines, Universidade de Lisboa, Lisbon, Portugal

**Keywords:** phenylketonuria, human induced pluripotent stem cells, organoids, neurodegeneration, neurodevelopment, disease modeling

## Abstract

Phenylketonuria is a recessive genetic disorder of amino-acid metabolism, where impaired phenylalanine hydroxylase function leads to the accumulation of neurotoxic phenylalanine levels in the brain. Severe cognitive and neuronal impairment are observed in untreated/late-diagnosed patients, and even early treated ones are not safe from life-long sequelae. Despite the wealth of knowledge acquired from available disease models, the chronic effect of Phenylketonuria in the brain is still poorly understood and the consequences to the aging brain remain an open question. Thus, there is the need for better predictive models, able to recapitulate specific mechanisms of this disease. Human induced pluripotent stem cells (hiPSCs), with their ability to differentiate and self-organize in multiple tissues, might provide a new exciting *in vitro* platform to model specific PKU-derived neuronal impairment. In this review, we gather what is known about the impact of phenylalanine in the brain of patients and highlight where hiPSC-derived organoids could contribute to the understanding of this disease.

## Introduction

Phenylketonuria (PKU; OMIM #261600) is a recessive genetic disorder of amino acid metabolism, affecting 1-5/10,000 individuals worldwide (ORPHANET, 2012). PKU is caused by mutations in the *PAH* gene encoding for phenylalanine hydroxylase (PAH). Human PAH is a cytoplasmic aromatic amino acid hydroxylase and is mainly expressed in the liver. It is involved in the first and rate-limiting step of L-phenylalanine (L-Phe) catabolism where about 75% of dietary L-Phe is degraded into H_2_O and CO_2_. Human PAH hydroxylates L-Phe into L-tyrosine (L-Tyr) in the presence of dioxygen and the cofactor tetrahydrobiopterin (BH_4_) (Mitchell et al., 2011; Flydal and Martinez, 2013). Impaired PAH activity leads to increased L-Phe concentrations in plasma (hyperphenylalaninemia; HPA) and brain, causing severe cognitive and neuronal impairment in untreated patients, sequelae in late treated individuals and neuropsychological symptoms in early-treated adult PKU patients who discontinue treatment (MacDonald et al., 2010; Ashe et al., 2019). For now, no curative therapy for PKU is available and restrictive diet, administration of the synthetic form of the PAH cofactor (acting as a pharmacological chaperone) or enzymatic substitution therapy with a pegylated form of the non-constitutive phenylalanine ammonia lyase (PEG-PAL), are the main options for PKU treatment, aiming to bring circulating L-Phe to levels lower than 360 μM in children, and 600 μM in adults, and closer to physiological values (≈120 μM) (Hafid and Christodoulou, 2015; Wegberg et al., 2017; Burton et al., 2020). It is presently well established that PKU treatment must be implemented as soon as possible, after birth, to avoid irreversible brain damage. However, and despite the diverse array of pharmacological and non-pharmacological alternatives, the available therapeutic options are not always as effective as needed, and the disease itself imposes a heavy social and economic burden for patients, their caregivers, and the society.

The central nervous system (CNS) is deeply affected in poorly or untreated PKU patients, with the hippocampus and cortex described as the most affected regions mainly due to the neurotoxic effect of L-Phe and the low levels of monoamine neurotransmitters such as serotonin, dopamine, adrenaline, and noradrenaline. L-Phe neurotoxicity and teratogenicity is also evident in the maternal PKU phenotype, where the fetus, even though not carrying two mutant *PAH* alleles, will develop cardiomyopathy, microcephaly, severe intellectual disability, and growth retardation due to high maternal levels of L-Phe during pregnancy (Levy and Ghavami, 1996; Cho and McDonald, 2001). Even in early and continuously treated PKU patients, suboptimal neuropsychological outcomes are frequently observed (Table 1), which indicates a chronicity induced by controlled, but not physiologically normal L-Phe levels (Bilder et al., 2017; Ashe et al., 2019). The understanding of these mechanisms will allow PKU patients to prepare for healthy aging. In fact, the first PKU patients who have benefited from early treatment are now entering middle age (fifth and sixth decade of life), and aging may represent an additional vulnerable period for PKU patients as they may still be more susceptible to develop neurodegenerative diseases than healthy people, given the chronic effects of L-Phe in the CNS.

**TABLE 1:** Phenylketonuria (PKU)-related neuropsychiatric symptoms and neurotransmitter deficiency as a function of treatment initiation/adherence.

	Non-PKU healthy adults	Untreated/Late-diagnosed adults	Early treated adults with poor adherence to treatment	Early treated and well-managed adults
**L-Phe plasma levels**	120–320 μmol/L	Of-diet/untreated: > 1200 μmol/L	Of-diet: > 600 μmol/L On diet: 120–600 μmol/L	120–600 μmol/L

**5-HIAA, 5-HTP, HVA and L-DOPA CSF levels**	5-HIAA: 60–130 nmol/L		5-HIAA: 13–77 nmol/L	
	5-HTTP: 6–12 nmol/L		5-HTP: 2–15 nmol/L	
	HVA: 120–400 nmol/L		HVA: 79–302 nmol/L	
	L-DOPA: 5–13 nmol/L		L-DOPA: 1–10 nmol/L	

**Neuropsychiatric symptoms**		Psychotic symptoms Autistic behaviors Hyperactivity Aggression Depressed mood Severe cognitive impairment	Anxiety Impaired visual-motor coordination Mood swings Visual-spatial and visual-constructive disabilities Fine-motor skills impairment Attentional problems Depressed mood	Depressed mood Social isolation/withdrawal Generalized anxiety Phobias Social maturity deficits Executive function impairment Low self-esteem

*5-Hydroxyindoleacetic acid (5-HIAA), 5-hydroxytryptophan (5-HTP), Homovanillic acid (HVA), metabolites from serotonin, tryptophan, and dopamine, respectively, and Levodopa (L-DOPA), a dopamine precursor, represent the most affected neurotransmitter pathways in PKU. Values depicted assume the maximum and minimum concentrations found in patients CSF in the available literature (McKean, 1972; Butler et al., 1981; Pilotto et al., 2018). No distinction was made between untreated and early diagnosed patients, as values were always within the defined ranges. Phenylalanine (L-Phe) plasmatic levels can be consulted in The Complete European Guidelines for Phenylketonuria. Adapted from Ashe et al. (2019).*

Despite the extensive number of studies on this topic, the exact pathophysiological mechanism of HPA neuropathology remains elusive and many questions still need to be answered. While PKU represents a monogenic disease, which initially results in the peripheral build-up of L-Phe, direct/indirect pathologic consequences are highly complex with limited molecular insights and, as a result, lack of effective therapeutic strategies. Like other brain diseases, existing knowledge on PKU is limited to those gained from *post-mortem* brain tissue, *in vitro* two dimensional (2D) experiments, and rodent PKU models. Despite the accumulated wealth of knowledge resulting from these studies, all of them face the incapacity to fully reproduce the complexity of human neurodevelopment and neurological processes. The advances in stem cell technologies, such as induced pluripotent stem cells (iPSCs), may contribute to the elucidation of the mechanism of PKU neuropathology, since iPSCs can differentiate *in vitro* into any of the germ layers and self-organize into 3D brain organoids, resembling the complex developing human brain. The establishment of strategies for differentiating non-neural tissue along with neural tissue also provides a platform for a more systemic understanding of diseases that affect the brain (Paşca, 2018; Koo et al., 2019). Additionally, through iPSCs, organoids can be derived from patients, which allows exploring inter-patient variabilities, with co-generated isogenic variants serving as controls (Soldner et al., 2011; Wang et al., 2014).

In this review, we provide an overview on the current knowledge of PKU neuropathology and discuss the opportunity that iPSCs represent in clarifying specific mechanisms involved in the reported mental and motor sequalae, by offering examples of engineered organoid models and their relevance in the context of this disease. One major constraint when modeling neuronal disorders with human iPSC-derived organoids relates to aging. The displayed phenotype is usually similar to the prenatal brain, and fully recapitulating aging processes that represent a major risk for the development of neurodegenerative diseases has been challenging. We will also convene how this limitation (among others) may represent a step-back on modeling possible predispositions for dementia in managed adults with metabolic diseases, and which solutions are being considered to overcome such limitations. Nonetheless, even 3D-brain models displaying a prenatal brain phenotype may provide information, at the molecular level, on how the developing brain is affected, and its connection to microcephaly (maternal PKU) and to the reported intellectual disability.

## Neuropathology of Phenylketonuria

In PKU, the reported mental arrest usually develops after birth since maternal PAH activity protects the fetus against higher L-Phe concentrations during embryonic development. Hence, the consequences of *PAH* mutations are compensated until birth. PKU treatment must then be implemented immediately after birth, usually by restriction of L-Phe dietary intake, as the impaired PAH function results in the accumulation of L-Phe. Neuropsychological sequelae associated with PKU have been linked to several biological mechanisms. In untreated/late-diagnosed PKU patients these include direct toxicity from L-Phe on myelination, leading to hypomyelination and gliosis of systems that normally myelinate late, progressive white matter degeneration (leukodystrophy), most commonly seen in adults, and developmental delay or arrest in the cerebral cortex, with atrophy of specific brain structures and impairment of functional neuronal connectivity (Dyer, 1999; Huttenlocher, 2000; Horling et al., 2015; Bilder et al., 2017). HPA results in multiple neurotransmitter and amino acid deficiencies in the CNS leading to a compromised brain homeostasis. At the cellular level, high L-Phe concentrations were shown to affect dendritic branching and decrease synaptic density, plasticity, and excitability (Hörster et al., 2006; González et al., 2016; Saudubra and Garcia-Cazorla, 2018). Hypomyelination, demyelination, oxidative stress damage and brain energy impairment are also recognized as drivers of PKU-associated neuropathology, being triggered by a direct neurotoxic effect of L-Phe and neurotransmitter deficiency (Pilotto et al., 2018; Vardy et al., 2019).

A wealth of research efforts aimed at elucidating the molecular mechanisms by which phenylalanine-induced neurodegeneration may occur have been made, but the precise details of this process remain elusive.

### Which Are the Pathways Affected by PKU in the Critical Period of Brain Development and Throughout a Patient’s Life?

**FIGURE 1:**
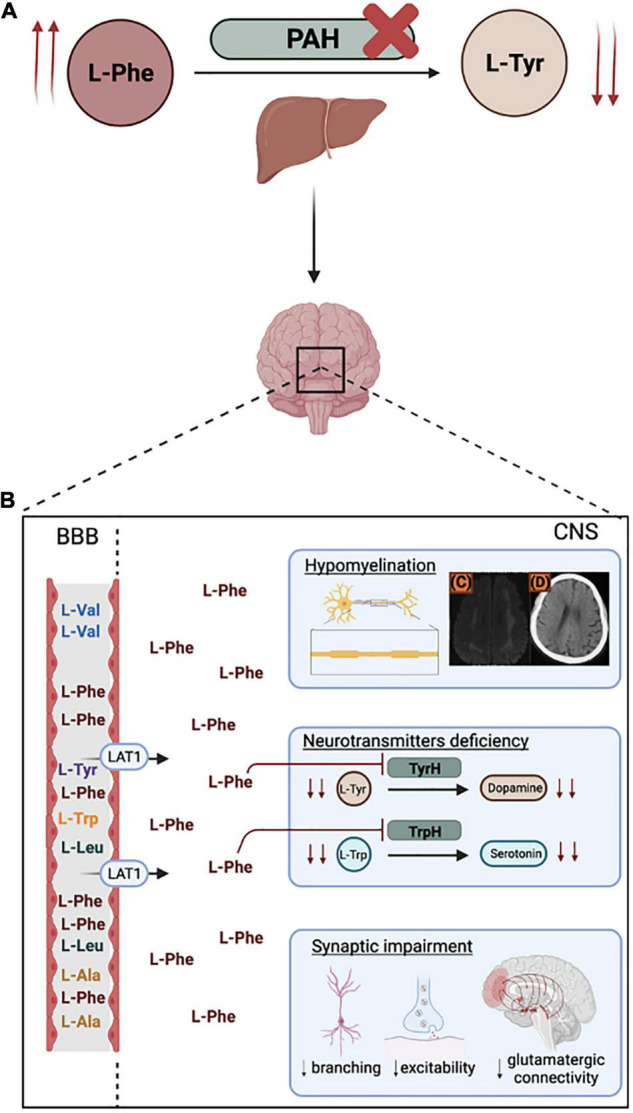
Phenylketonuria (PKU) pathophysiology. **(A)** Metabolic impairment of phenylalanine hydroxylase (PAH) in the liver leads to increased plasmatic phenylalanine (L-Phe) (hyperphenylalaninemia) and decreased tyrosine (L-Tyr). **(B)** At the Blood Brain Barrier (BBB), L-Phe competes with higher affinity for the L-type amino acid transporter (LAT-1), leading to neurotoxic L-Phe levels in the Central Nervous System. Hyperphenylalaninemia negatively impacts myelinating processes [**(C)** and **(D)** represent low-density whyte matter lesions and temporal/frontal lobes atrophy in a late-diagnosed child (adapted from Chen et al., 2019)] and leads to neurotransmitters deficiencies and synaptic impairment. Figure created with BioRender.com.

In the context of PKU, the so-called vulnerable period extends after birth until the completion of brain development. This means that processes such as myelination, synaptogenesis, and synaptic pruning, which are completed long after birth, are highly susceptible to extracellular environmental factors. For some prefrontal areas, this represents an at-risk period until adolescence (Paus et al., 1999; Huttenlocher, 2000; Budday et al., 2015). Untreated and late diagnosed patients are a clear example of impaired brain development, triggered by a direct neurotoxic effect of L-Phe exposure, and by a generalized neurotransmitter deficiency. Normal brain function depends on the regulated inflow of nutrients through the Blood Brain Barrier (BBB). In the CNS, monoamine neurotransmitters are synthesized upon uptake of the necessary precursors, such as the large neutral amino acids (LNAAs) L-Tyr and L-tryptophan (L-Trp). The LNAAs are transported into the CNS by the L-type amino acid transporter (LAT-1). In supraphysiological L-Phe concentrations, carrier saturation and competitive inhibition lead to reduced uptake of the remaining LNAAs into the brain (Shulkin et al., 1995; Pietz et al., 1999), including L-Tyr and L-Trp, the precursors of catecholamines (dopamine, adrenaline, and noradrenaline) and serotonin, respectively (Figure 1). Evidence from classical-PKU mouse model (*Pah*^enu2^) has shown that decreased influx of LNAAs into the CNS leads to a biogenic amine deficiency and has downstream effects on cerebral protein synthesis, affecting not only neurotransmitter synthesis but also myelinating processes, and pre- and post-synaptic processes implicated in the formation and maintenance of synapses during brain postnatal maturation (Castillo et al., 1988; Pascucci et al., 2002, 2008; Imperlini et al., 2014; González et al., 2016). These findings correlated with reports on untreated PKU patients having severe impairment of brain architecture and maturation: aside from abnormalities in myelination, also cell density, cell organization, and dendritic arborization were altered, together with fewer synaptic spines, dendritic processes in Purkinje cells, reduced soma size in neurons and neuronal loss (Dyer et al., 1996).

A correlation between plasma concentration of L-Phe and white-matter lesions was previously demonstrated in PKU mouse models (Dyer et al., 1996). Given that oligodendrocytes (OLs) are major players in myelinating processes, it should be expected that this cell population may be specifically vulnerable to L-Phe induced toxicity. Interestingly, Schoemans et al. (2010) demonstrated that the number and viability of OLs from PKU mouse models and cultured cells was not affected by high plasma levels of L-Phe. Indeed, prior to these studies, Dyer et al. (1996) had shown that white matter from PKU affected cerebrum and cerebellum (human and mouse) was gliotic, but that the OLs were viable. These OLs were, however, unable to produce myelin, leading to an altered phenotype from myelinating to non-myelinating and resulting in severe myelination delays (Dyer et al., 1996). This mechanism might be valid for untreated or late diagnosed patients, but for early treated patients myelination is thought to proceed normally during development. Therefore, the chronic demyelination that is observed in early diagnosed and continuously treated patients, might instead be linked to degradation of previously myelinated structures, and its severity and reversibility may be related to dietary adherence and metabolic control profile (Dyer et al., 1996; Hörster et al., 2006; Anderson and Leuzzi, 2010; Schoemans et al., 2010; Bilder et al., 2017; Ferreira et al., 2021).

One striking evidence of L-Phe neurotoxicity during neurodevelopment stages is the maternal PKU phenotype. Phenylketonuric women, normally fertile, have high risk pregnancies with offspring being born non-PKU, but with intrauterine growth retardation (IUGR), intellectual disability, microencephaly and congenital heart disease. Like the uptake in the brain, phenylalanine shares active placental transport with other neutral amino acids. Thus, high levels of circulating maternal L-Phe are likely to result in inhibition of other amino acid transport, by competition for their carriers (Levy and Ghavami, 1996). Hence, a positive L-Phe gradient into the placenta leads to exposure to 1.5-2 times the phenylalanine concentration of maternal blood, imposing an even greater risk for the developing fetus than the one expected based on maternal L-Phe levels (Schoonheyta et al., 1994; Cho and McDonald, 2001). It is also known that hyperphenylalaninemia embryopathy features hypoplasia of corpus callosum and cerebral atrophy, to which severity is directly proportional to the timing and proper management of the levels of maternal L-Phe (Levy and Ghavami, 1996; Graf et al., 2013). However, the exact etiology of maternal PKU still lacks clarification and consensus. Studies conducted in rodent models of the disease have suggested that both HPA and exposure to high levels of the L-Phe metabolite, phenylacetate, were sufficient to induce a maternal PKU phenotype in non-PKU offspring (Loo et al., 1984; Levy and Ghavami, 1996; Zeile et al., 2018). However, examination of human fetal tissues on termed pregnancies did not show high levels of L-Phe metabolites in the fetus brain. Also, a woman with a metabolic disorder that induced the accumulation of high levels of L-Phe toxic metabolites, but not L-Phe, gave birth to normal offspring (Levy and Ghavami, 1996). *Post-mortem* delay is not reported but could affect such observations (Poloz and O’Day, 2009; Zissler et al., 2020). Additionally, this apparent contradiction might also be explained by differences in CNS development between higher primates and rodents (Shedlovsky et al., 1993).

### Hyperphenylalaninemia Affects Cellular Mechanisms

While it is possible that L-Phe metabolites induce the toxic effect associated with a PKU-phenotype, alternatively, a direct effect of HPA on downstream processes and products of L-Phe conversion to L-Tyr may occur. Besides leading to decreased bioavailability of other LNAAs, high brain L-Phe levels inhibit the activity of L-Tyr and L-Trp hydroxylases, both rate-limiting enzymes for the synthesis of dopamine and serotonin, respectively (Guttler and Lou, 1986; González et al., 2016; Figure 1). These deficits have been reported not only in untreated patients and classical-PKU mouse models (Puglisi-Allegra et al., 2000), but also in well managed adults (Giovannini et al., 1988; Landvogt et al., 2008; Pilotto et al., 2018) and correlated with brain damage and atrophy of specific cortex areas (Landvogt et al., 2008; Pilotto et al., 2018). Curiously, the serotonin axis appears to be more susceptible than dopamine, as inhibition of dopamine synthesis is only significant at L-Phe concentrations three times higher than those needed for serotonin synthesis inhibition. This difference might explain why serotoninergic related symptoms, such as anxiety, depression, mood swing, aggressiveness, etc., are more common in PKU patients than dopaminergic ones (Pilotto et al., 2018).

Multiple reported observations suggest an effect of L-Phe levels on synaptic transmission and synaptogenesis (Figure 1). Synaptogenesis impairment has been observed in primary mouse cortical neurons upon *in vitro* exposure to L-Phe, and this effect has been associated with decreasing activity of the glycolysis enzyme pyruvate kinase (Costabeber et al., 2003). Rat PKU models showed reduction of GABA inhibitory effect (involved in cognitive processes), due to decreased dendritic basilar processes of large pyramidal cells from deep cortical layers (Hörster et al., 2006). Synaptic plasticity in the PKU mouse hippocampus is also affected, with major impact on pre- and post-synaptic amplitude and frequency. Decreased synaptic plasticity and the observed glutamatergic synaptic impairment are thought to be related (Diering and Huganir, 2014; Barnes et al., 2020). High L-Phe levels seem to induce differential expression of the neurotransmitters N-methyl-D-aspartate (NMDA), involved in learning and memory, and α-amino-3-hydroxy-5-methyl-4-isoxazolepropionic acid (AMPA), with a role in spatial learning, receptor subunits by competition for their glycine and glutamate binding sites, respectively. Expression of glutamate receptor mGlu5 is also altered in the *Pah^enu2^* mice, a mouse model carrying a single amino acid substitution that severely inhibits normal PAH activity reminiscent of PKU (Glushakov et al., 2005; Simões et al., 2007; Martynyuk et al., 2010; Morris, 2013; Imperlini et al., 2014; Horling et al., 2015; Nardecchia et al., 2018). As glutamatergic transmission is selectively depressed at a range of L-Phe concentrations (observed in the brains of PKU patients) by a combination of pre- and post-synaptic actions, the cognitive arrest observed in PKU patients could be a consequence of the disruption of this pathway.

Overall, there is an extensive body of literature exploring potential mechanisms for L-Phe induced neurotoxicity. Protein expression profiling in the *Pah*^enu2^ mouse brain pinpointed neuron differentiation, dendritic growth/branching, oxidative metabolic pathways, and cerebral glucose metabolism as the most affected cellular processes (Imperlini et al., 2014). However, it is still unclear which are the mechanisms directly related to high L-Phe exposure, and which are due to cause-effect of impairment of high complex brain processes. To add to this challenge, it is important to highlight that these processes involve multiple cell layers and are highly dependent on the model animals under study.

### Phenylalanine Mediates Oxidative Stress

The brain has a relatively low antioxidant defense capacity and high lipid (unsaturated fatty acids) and catecholamine content, which are highly susceptible to reactive oxygen species (ROS) attack. Oxidative stress has been observed in some inborn errors of metabolism in general, due to the accumulation of toxic metabolites and restrictive diets that alter the antioxidant status (Rocha and Martins, 2012; Stepien et al., 2017). Several PKU-disease model and proteomic studies have suggested multiple impairments in the brain antioxidant defense system, brain energy metabolism (tricarboxylic acid cycle and glycolysis) and reactive oxidative species (ROS) production and signaling (Sirtori et al., 2005; Imperlini et al., 2014; Bortoluzzi et al., 2021). Exposure of cultured newborn rat astrocytes to L-Phe resulted in the generation of free radicals inducing oxidative stress and reduced astrocytic viability (Preissler et al., 2016). Rat-derived hippocampal and cerebral cortex regions showed that phenylalanine exposure induced oxidative stress by decreasing the levels of reduced glutathione, or GSH, which is an important regulator of antioxidant defense mechanisms (Fernandes et al., 2010). It has been suggested that administration of antioxidant therapeutics may alleviate PKU-related symptoms (Martinez-Cruz et al., 2002), given that oxidative damage could be prevented by the addition of antioxidants and free radical scavengers in the aforementioned studies (Feksa et al., 2002; Fernandes et al., 2010; Preissler et al., 2016). However, while some data suggests beneficial effects of antioxidant supplementation, other clinical studies argue against it (Mazzola et al., 2013). While oxidative stress in the PKU patient brains seems a straightforward assumption, judging from the limited antioxidant capacity of the brain in general, peripheral oxidative stress status of PKU patients and observations from brains of PKU rodent models, no reports are known to date that demonstrate brain oxidative stress status in PKU patients. Indeed, correlation between antioxidant status and blood L-Phe levels in PKU patients has also been challenging to obtain, explaining the number of patients with good dietary adherence that still present neuronal damage (Rausell et al., 2019). Data that support such hypothesis include the observation of cerebral glucose hypometabolism in eleven adult PKU patients (McGinnity et al., 2021) which is known to scale with oxidative stress in preclinical Alzheimer’s disease (AD) patients (Mosconi et al., 2008). Therefore, oxidative stress might be an important neurodegeneration-inducing mechanism in PKU, possibly contributing to the neuropathology of late onset neurological and psychological impairments observed even in early diagnosed and uninterruptedly treated PKU adults. There have been attempts to define oxidative stress biomarkers reliable enough to correlate to neuronal damage in PKU patients (Bedin et al., 2001). While peripheral oxidative stress has been observed in PKU patients, indicated by elevated plasma marker levels for oxidative stress (reviewed in Rausell et al., 2019), only a handful of studies investigated brain-localized oxidative stress in PKU. Using a rat model, L-Phe as well as L-Phe catabolic by-products phenylpyruvate and phenylacetate increased specific markers for lipid peroxidation (TBA-RS) and protein oxidative damage, both in the cerebral cortex and the cerebellum (Fernandes et al., 2010). PKU-related brain oxidative stress has also been demonstrated in rat (Kienzle Hagen et al., 2002; Martinez-Cruz et al., 2002; Moraes et al., 2010, 2013; Rosa et al., 2012; Simon et al., 2013) and mouse PKU models (Wyse et al., 1995; Ercal et al., 2002; Sirtori et al., 2005; Fernandes et al., 2010). Nevertheless, little information regarding more CNS-specific oxidative stress biomarkers have been reported in PKU patients (Rausell et al., 2019).

### What Are the Consequences of PKU for the Aging Brain?

Nowadays, due to the worldwide implementation of large-scale newborn screening programs and the early initiation of treatment, severe neurological and neurocognitive impairments resulting from PKU have been positively ameliorated or even prevented (Susi and Guthrie, 1963; Bilder et al., 2017). What is astonishing is that early diagnosed PKU patients, who maintained a well-controlled restrictive diet during childhood, do not appear to be free of sequelae (Ashe et al., 2019). This observation probably reflects the fact that management of L-Phe levels for these patients becomes increasingly difficult throughout life, as pointed out by recent surveys (Walter et al., 2002; MacDonald et al., 2010; Brown and Lichter-Konecki, 2016). For PKU adults with L-Phe metabolic levels above the upper limit threshold, cognitive impairments are more apparent and associated with anxiety, hyperactivity, mood swings and even motor dysfunction (Landvogt et al., 2008; Romani et al., 2017). Therefore, variability in adherence between individuals and throughout life seems to be one of the main reasons for different cognitive outcomes in early treated PKU patients (Bilder et al., 2017; Romani et al., 2017; Table 1). It has been reported that early-treated PKU adults have reduced performance in processing speed and complex executive functions, although maintaining relatively normal skills in learning, spelling, naming, verbal, and visuospatial memory (Ashe et al., 2019). As life expectancy of PKU patients is similar to that of healthy individuals, and even in healthy individuals aging coincides with progressively increased oxidative stress levels (Kandlur et al., 2020), impaired protein homeostasis and failing protein quality control systems (Higuchi-Sanabria et al., 2018), this may represent an additional vulnerable period for PKU patients. Given that well-managed PKU patients are still exposed to higher L-Phe plasmatic levels than healthy individuals, and adherence is most of the time not consistent, it is possible that PKU individuals may be more sensitive to aging processes. For instance, is protein quality control systems age-related degradation exacerbated by the PKU brain environment? Does aging-related neuronal degeneration start earlier in a PKU patient when compared to a healthy person? It is now accepted that severity of neuronal damage is related to how early and how well metabolic management is imposed. To what extend unstable compliance and acute or chronic exposure to pathological L-Phe levels throughout the patients’ lifespan will result in specific neuropathological outcomes is still to be determined.

Neurotransmitter and synaptic dysfunction, neuronal damage, oxidative stress, and energy metabolism impairments, among others, are also associated with age-related neurodegenerative processes (and thus neurodegenerative diseases) (Bossy-Wetzel et al., 2004; Jellinger, 2010). Indeed, several studies have showed the presence of elevated brain L-Phe levels (without genetic impairment of PAH) in a subset of patients with AD, associated with symptomatology exacerbation and neuroimmune activation (Neurauter et al., 2008; Wissmann et al., 2013; Corso et al., 2017). Another striking evidence of a possible neurodegenerative etiology in PKU has been reported by Adler-Abramovich et al. (2012), showing that L-Phe assembled in cytotoxic organized amyloid fibrils, that can assemble into plaques in *Pah*^enu2^ mice brain and *post-mortem* PKU patient parietal cortex. This assembly was found to be L-Phe dose-dependent and accompanied with glia cell infiltrations (Adler-Abramovich et al., 2012). These Phe-amyloid-fibrils were also able to cross-seed soluble globular proteins, at physiological concentrations, *in vitro* (Anand et al., 2017). As PKU induces long-term dopaminergic deficits, it could also lead to neuropathogenesis similar to that of Parkinson’s disease (PD) (Velema et al., 2015). However, even with *Pah*^enu2^ mice studies showing a PD-like neurodegenerative injury in nigrostriatal dopaminergic neuros (Emburya et al., 2007), the parkinsonism reported in some late-treated adults and motor dysfunction in untreated PKU patients is thought to be directly related to hypo- and demyelination, and secondary to dopamine deficiencies (Limphaibool et al., 2018).

Currently, the first early treated patients are middle-aged. Adulthood may represent an additional vulnerable time for PKU patients, as the compensation mechanisms for L-Phe accumulation in the brain may be impaired by normal brain aging processes. Indeed, brain-localized clearance pathways alternative to peripheral PAH are limited. Transamination is one of the most prominent alternatives to eliminate excessive levels of L-Phe in the brain (George and Gabay, 1968). While upregulation of this enzyme seems an interesting option to maintain L-Phe levels within strict threshold levels, it leads to accumulation of phenylpyruvate and phenylacetate, which, at elevated levels, exert toxic activity to cells of the brain ([Bibr B112][Bibr B112], 1973; Sutnick et al., 1974). Therefore, even if well managed, chronic exposure to L-Phe could have progressive impact across the patient’s lifespan and potentially lead to progressive neurodegeneration mechanisms (Romani et al., 2017; Ashe et al., 2019). Consequently, a greater susceptibility to age-related neurodegenerative diseases should be contemplated in PKU patients. Some of the observed disruptions naturally occurring in healthy aging individuals could then emerge at a younger age in early treated adults with PKU (Sirtori et al., 2005; Pilotto et al., 2018; Vardy et al., 2019). To which degree of exposure to high L-Phe levels, chronic or acute, and poor dietary compliance at some point of a patient’s life, impacts such predisposition is still to be determined.

## Using Human Organoid Models to Understand PKU-Driven Pathophysiology of Brain Neurodegeneration

In the past decade, human Pluripotent Stem Cell (hPSC) technology has been widely used to study human biological processes and diseases. hPSCs can self-organize in tissue/organ-like structures, recapitulating human-specific molecular processes and pathways, in the presence of cell-to-cell and cell-to-matrix signaling. Based on this ability to self-organize, experimental cultured systems have been developed and improved to mimic multicellular organization of different tissues *in vitro* (Paşca, 2018; Koo et al., 2019). Whereas the study of human diseases was hindered by ethical issues in human experimentation, and thus lack of accurate human-like models, now human-live samples are simpler to access, through patient-specific iPSCs. 3D-models are thus much easier to obtain, allowing for the development of human-like disease models against the patient genetic background. Building 3D organoid structures using iPSC technology could then be seen as an alternative to approach the limitations imposed by the human brain complexity (Hartlaub et al., 2019; Koo et al., 2019). Studies on neurodegenerative diseases and neurodevelopmental disorders using such 3D-models have already demonstrated their utility, showcasing clear differences between healthy and diseased phenotypes (Gonzalez et al., 2018; Mehta et al., 2018; Kim et al., 2019; Koo et al., 2019). Hence, given the complexity of PKU pathogenesis, brain organoids could provide an appropriate solution to target a specific set of weaknesses found in the traditional experimental set-ups.

### We Need a Human(ized) Model to Understand Relevant Molecular Parameters Driving PKU

The mechanisms causing the intellectual disabilities triggered by PKU-derived hypomyelination/demyelination processes, neurotransmitter deficiencies, oxidative stress and synaptic plasticity and connectivity, affect multiple brain regions and their interconnectivity. Until now, most studies on PKU were based on cell lines, mouse PKU models or *post-mortem* tissues from PKU patients. Despite the accumulated knowledge resulting from these studies, they face several limitations in their capacity to fully reproduce the intricacies of human neurodevelopmental processes: human *post-mortem* or surgically removed samples have variable genetic and environmental backgrounds and are limited to end-point analyses (Martynyuk et al., 2010; Grenier et al., 2020) and 2D cell models lack important cell-to-cell and cell-to-extracellular matrix interactions, which are crucial for the regulation of several neurodevelopmental stages (Chiaradia and Lancaster, 2020). Specific fundamental aspects of neurodevelopment are conserved in mammals, enabling direct translation of the large amount of knowledge accumulated from studying animal models to humans (Zhao and Bhattacharyya, 2018). However, there are human-specific characteristics of brain development that lead to highly complex structures and thus higher-order functions. Consequently, when modeling human neurodevelopmental disorders, not all human disease mechanisms can be recapitulated using an animal model. The most apparent difference is the larger size of the human cortex. Specific cellular and mechanistic characteristics are unique to primates. For instance, during neurodevelopment, the inner fiber layer and outer subventricular zone, extremely important for allowing neuronal output expansion in humans, are both absent in mice (Lancaster et al., 2013). Moreover, human neurodevelopment takes place during a longer gestational period, and is prolonged and pruned throughout childhood and adolescence (Zhao and Bhattacharyya, 2018). This is more evident in the case of white matter development, which is progressive up to the age of 22 years old in humans (Chanoumidou et al., 2020). Human cortex processes are also highly dependent on multiple classes of interneurons, when compared to other mammals (Zhao and Bhattacharyya, 2018). Regulatory elements such as cell fate and proliferating signaling, and genetic expression differences in synaptic transmission and plasticity can contribute to a human-specific phenotype (Koopmans et al., 2018; Zhao and Bhattacharyya, 2018; Lee et al., 2020). Regarding the classical-PKU mouse model (*Pah*^*enu*2^), while practical from the metabolic PAH deficiency perspective, it presents limitations when used for studies on the neurological symptoms. Firstly, the genetic BTBR—“Black and Tan Brachyury”—background mice strain of the *Pah*^enu2^ mouse model, leads to corpus callosum agenesis and hippocampal commissure defects, which are not seen in PKU patients, and thus can obscure the behavior assessment of PKU-related defects (Bruinenberg et al., 2016). Also, it has been reported that LAT-1 expression in the BBB is higher in rodents than in humans, which could mean that transport of LNAAS in humans through this transporter might be overestimated (Uchida et al., 2011; Hoshi et al., 2013). All these aspects have contributed to conflicting results between different model paradigms. Thus, additional studies using advanced humanized culture models should be performed. Genetic manipulation of organoids is an exciting strategy to obtain insights into the various hypothesized molecular pathways that play a role in PKU pathology. With the development of CRISPR-Cas tools, gene editing of mammalian cells has become accessible (Cong et al., 2013; Mali et al., 2013). One of the most straightforward PKU-related targets that may be interrogated using gene editing approaches include PAH knock-out or mutations in a liver organoid model (Cao et al., 2021). We can also modulate and analyze other PKU-related targets.

Brain organoids have only recently been exploited to address oxidative stress mechanism-related questions. The relatively limited access to PKU patient brain material makes a brain organoid model capable to recapitulate key aspects of PKU-related oxidative stress particularly appealing. The proposed impact of high brain L-Phe levels on lipid peroxidation or DNA oxidative damage can be reproduced using such models by exposing brain organoids in culture to supraphysiological L-Phe concentrations and measuring oxidative stress-related markers. These markers can extend from DNA damage, lipid peroxidation, protein oxidation products, or changes in cellular homeostatic mechanisms including unfolded protein response (Paşca et al., 2019). In the past, a hypoxia-induced oxidative stress model of neurovascular unit derived from stem cells demonstrated that cellular metabolic rate, measured by mitochondrial ATP production, inflammation and BBB-permeability were affected as a function of low oxygen levels (Nzou et al., 2020). Three-dimensional stem cell-derived human cortical spheroids have been used to demonstrate the effects of oxidative stress, as a function of hypoxic (<1% O_2_) conditions, in cell death and disruption of protein homeostasis, to model hypoxic encephalopathy of prematurity (Paşca et al., 2019). Similarly, HIF-1α, the generic hub regulator of oxidative stress pathways (Jaakkola et al., 2001), may be knocked-down (or -out) to dissect the role of brain oxidative stress in PKU-related neuronal cell death. On the other hand, L-Phe transamination enzymes could be up-regulated to induce brain L-Phe clearance and further provide insights into the role of L-Phe by-products in oxidative stress.

Manipulation of downstream regulatory enzymes of L-Phe conversion (e.g., tyrosine hydroxylase), involved in specific neurotransmitter synthesis (like dopamine) and that have reduced expression levels in the brains of *Pah*^enu2^ PKU mouse models (Joseph and Dyer, 2003; Emburya et al., 2007), could also allow a better understanding on the neurotransmitter dysfunction reported in PKU.

### We Need Multiple-Cell Type Containing Models to Fully Recapitulate PKU Pathophysiology

Due to the suggested multicellular involvement of HPA in PKU-driven neuronal damage, disease models designed to push forward our understanding of pathological mechanisms should incorporate the various cell types typically present in the brain, including glia and neurons, at relevant ratios. The personalized iPSC technology enables further studies of disease-related cellular processes, reproducing the inter-patient variability observed in PKU (for review on PKU genotype/phenotype relation see Hillert et al., 2020). Region-specific organoids allow insights on organogenesis and on the consequences of a toxic environment (such as exposure to high L-Phe levels) during neurodevelopment of specific neurons, as they mostly reach mid-fetal stages (Paşca, 2018). Derivation of specific CNS regions can now be achieved through two main approaches: directed differentiation, which is highly dependent on cell signaling manipulation to direct the culture toward the required brain region cell types, and spontaneous differentiation, where 3D cultures lacking inductive signaling lead to a multitude of neuronal cell types and sub-specification layers within the same spheroid (Lancaster et al., 2013; Sakaguch et al., 2015; Chang et al., 2020; Chen et al., 2020).

As neurodevelopmental disorders affect different brain regions and their intercommunication, fused organoid systems enable the formation and analysis of more complex inter-region neuronal circuitry. These fused organoid models (assembloids) have already been established to study interneuron migration (Bagley et al., 2017) and are being used to modulate neurodevelopmental disorders such as Autism Spectrum disorders (Villa et al., 2021), Down-Syndrome (Xu et al., 2019) and Rett-Syndrome (Gomes et al., 2020). In the context of PKU induced neuropathology, where forebrain, hippocampus, striatum (Miura et al., 2020), and their inter-connections are affected, assembloids represent a simplistic, yet viable, technology for understanding the underlying mechanisms.

On the other hand, given the complexity of brain processes and their tight relationship with peripheral signaling, there is an effort being made to incorporate brain microenvironment players within 3D brain models. For PKU neuropathology, glial cells and BBB are important factors that should be added to organoid models. Recent attempts to engineer vascularized models exhibited complex vessel networks, larger sizes, and lower levels of necrotic core formation, and were also able to mimic some aspects of the BBB, as confirmed by the expression of tight junctions, astrocyte and pericyte markers (Cakir et al., 2019; Chang et al., 2020). Additionally, blood perfusion within the organoid can be achieved in two distinct ways: grafting organoids into immunodeficient animals, or by using microfluid systems, known as organ-on-a-chip (Mansour et al., 2018; Matsui et al., 2021). The latter, if combined with fused brain-vascular organoids, is expected to be relevant for studying BBB events in humans, and consequently human-specific expression profiles (Matsui et al., 2021). The event of L-Phe uptake into the brain by LAT-1-mediated transport is one of them. Several attempts have been made to reverse the concentration gradient of L-Phe through supplementation with other LNAAs (Pietz et al., 1999; Spronsen et al., 2010), but Phe brain levels in PKU animal models remained significantly higher when compared to severe L-Phe dietary restriction controls (Vliet et al., 2019). This outcome still needs clarification, particularly when considering the LNAAs BBB-transport mechanisms. A brain-vascular organoid system could benefit PKU studies on LNAA transport through the BBB and elucidate some of these unresolved issues. For example, LAT-1 blood-brain barrier models, based on co-culture of astrocytes and brain endothelial cells, to manipulate preferential transport of one amino acid over another is an interesting strategy to study LAT-1-driven pathways of L-Phe brain accumulation.

**FIGURE 2:**
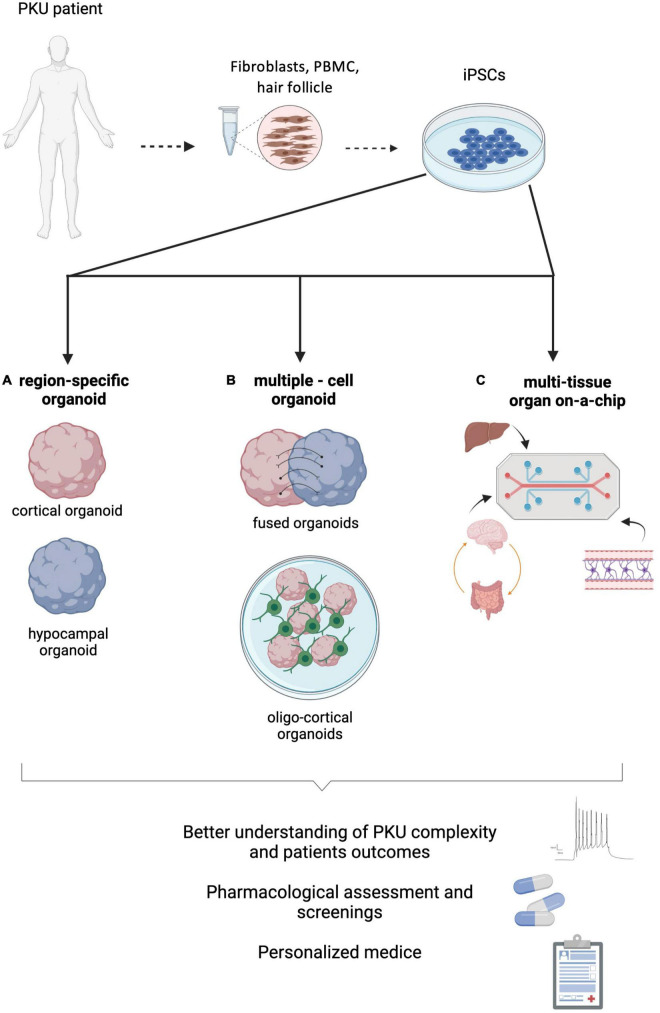
Phenylketonuria (PKU) modeling strategies using hPSCs derived from patients. **(A)** Region-specific brain organoids could help clarifying specific processes directly affected by the neurotoxic effect of phenylalanine (L-Phe). **(B)** Multiple-cell models, such as fused-organoids or oligo-cortical organoids, might shed some light on how inter-region connectivity or myelination are impaired by hyperphenylalaninemia. **(C)** Multi-tissue organ-on-a-chip systems, combining the main contributors to PKU neuropathology (brain, BBB, gut and liver) will enable understanding on the systemic complexity of this disease. Figure created with BioRender.com.

The acute hypomyelination, the later-onset chronic demyelination observed and their relationship with HPA have also been studied throughout the years. However, it remains to be clarified how OLs undergo the supposed phenotype switch from myelinating to non-myelinating, and how it affects glial plasticity. Questions such as if this mechanism is exclusive to acute hypomyelination observed in untreated/late-treated patients or which mechanisms lead to chronic demyelination in non-compliant adults are still to be answered. OL-enriched 3D-brain organoids displaying OL migration, myelinating markers, and wrapping processes around neuron axons have been developed by several groups (Madhavan et al., 2018; Marton et al., 2019; Shaker et al., 2021). Although the myelinating phenotype obtained is mostly identical to the later stages of fetal human third trimester, these models are also able to recapitulate monogenic leukodystrophy diseases (Madhavan et al., 2018; Marton et al., 2019; Shaker et al., 2021). As the PKU-derived hypomyelination in untreated patients is seen as a non-genetic leukodystrophy, it would be interesting to adapt these models to modulate the L-Phe neurotoxicity to the myelinating process. The current oligo-cortical organoid models might be appropriate to help us answering questions regarding the direct effect of high L-Phe levels on OL development and on myelinating OLs (Figure 2). Exposure to pathological L-Phe enriched medium, or knock-down of the enzyme 3-hydroxy-3-methylglutaryl coenzyme A reductase (HMGR), which regulates the rate-limiting step in the synthesis of cholesterol, an important component of myelin lipid (Shefer et al., 2000), are examples on how oligo-cortical organoids might allow studying the PKU-associated reduced myelination.

## Development of Multi-Tissue Models to Capture the Systemic Complexity of PKU

Despite the hepatic origin of PAH deficiency, its primary manifestation occurs at the neuronal level. The PAH-induced impairment in converting L-Phe into L-Tyr in the liver is dependent on the underlying *PAH* gene mutations (genotype), and to date, more than a thousand different mutations have been identified in the *PAH* gene, with most patients being compound heterozygous (Gersting et al., 2008). Consequently, the disease phenotype, and thus the severity of the intellectual arrest, is highly variable. Although extremely valuable, animal models are unable to recapitulate the extensive variability of the phenotypes associated with the disease, hindering pharmacological screenings and the development of potential therapies. iPSC-derived organoids enabled significant advances on disease modeling technologies, which have opened the doors to potentially understand organ/region-specific disease mechanisms that so far remained enigmatic (Skardal et al., 2017). Nonetheless, single organoid models are incapable of reproducing physiological multi-organ interactions that normally occur in the human body and are highly involved in disease settings such as the liver-brain connection in PKU. Thus, a systemic approach should be followed, to access key mechanisms and build even more complex disease models (Skardal et al., 2017; Picollet-D’hahan et al., 2021). Even though multi-organ-on-a-chip technology is taking its first steps, such approach has already been applied to model inflammatory bowel disease and ulcerative colitis (Trapecar et al., 2020), and diabetes mellitus type 2 (Bauer et al., 2017).

Considering that PKU is regarded as a protein misfolding disease, research on pharmacological chaperones as a therapeutic option has been growing in significance. However, the development of such therapies has been hindered by the heterogeneity of patient genotype and phenotype (Ho and Christodoulou, 2014). Current disease models have been struggling to overcome this question, which hampers clinical and pharmacological validation. In fact, the high number of PAH variants is reflected in the broad spectrum of metabolic and enzymatic phenotypes that lead to different disease severities, from non-PKU HPA to the most severe form of classical-PKU (Gersting et al., 2008; Underhaug et al., 2013). Consequently, predicting patient outcomes and developing successful pharmacological strategies has been challenging. Liver organoids have already been engineered for other genetic diseases (for full review see Cotovio and Fernandes, 2020) and recently fused with other organoids from different organ-systems (Koike et al., 2021). By taking advantage of the multi-tissue-organoid technology, modeling the liver-brain axis (the two major players of this disease) could represent a new framework, not only to shed some light on how cellular processes are differently affected between patients, but also to predict the efficacy of new therapies focused on ameliorating the enzymatic function and how well the neuronal processes responded to such therapies. For example, the BMN 307 investigational product from BioMarin Pharmaceutical (AAV5-PAH gene therapy), which already reached phase I/II clinical trials, was suspended due to adverse events observed in the mouse model (predisposition to develop malignancy) which, however, are not usually observed in larger animals or humans (Biomarin Pharmaceutical Inc., 2021). In this perspective human hepatic organoids would constitute an attractive tool to gain further insights into these potentially curative therapies. Organoids could be derived from patient iPSCs or after gene editing to generate specific variants. Indeed, a patient-derived, organoid-based, high-throughput drug screening system could be engineered to model treatment responses to conventional and developing treatments, possibly helping to decrease the number of pre-clinical trial failure. Also, the addition of circulating immune cells in the vascular channels would be helpful to predict adverse effects (Armstrong et al., 2021; Plebani et al., 2021) regarding therapeutic options, such as enzyme substitution by administration of pegylated phenylalanine ammonia lyase (PEG-PAL), known to have significant immunogenicity (Hausmann et al., 2019). Combinations between healthy- and PKU-derived liver and brain organoids could also be a strategy to modulate the interplay between the metabolic impairment and neuronal damage. Either way, such approaches would result in platforms that would allow to interrogate the effects of the heterogeneity observed in patients to the outcome of the disease.

Likewise, the brain-gut axis could be better understood by modeling it through multi-tissue organoids (Figure 2). It is nowadays accepted that there is a bidirectional relationship between the CNS and gut microbiota, with complex molecular mechanisms and anatomical connections involved, influencing behavior and cognitive processes (Tilocca et al., 2020). Moreover, evidence of a relation between dysbiosis and neurodegeneration has been accumulating (Raimondi et al., 2020), with impact on diseases such as PD (Sampson et al., 2016; Romano et al., 2021), AD (Vogt et al., 2017), Autism Spectrum disorders (Vuong and Hsiao, 2017) and anxiety/depression (Foster and McVey Neufeld, 2013; Capuco et al., 2020). Additionally, evidence of the impact of gut microbiota in post-natal neurodevelopment has been demonstrated in germ free animal models (Quigley, 2017). Diet-induced alterations in the gut microbiome can occur rapidly and induce dysbiosis (Alou et al., 2016; Martinez et al., 2021). Changes in amino acid composition and abundance can affect amino acid-metabolizing bacterial communities, modulate immune and antioxidant responses, and impair important pathways such as the glutathione pathway (for extensive review see Ma and Ma, 2019; Verduci et al., 2020). Therefore, a Phe restrictive regimen, imposed from birth throughout life, and most of the times leading to a chronic reduced consumption of amino acids, can promote a decrease in gut biodiversity. Indeed, studies have showed a decreased diversity in gut microbiome populations in PKU patients, observed even during childhood (Oliveira et al., 2016; Bassanini et al., 2019). Although the majority of PKU patients may experience neuropsychiatric comorbidities, that have been associated with dysbiosis, the real impact of gut imbalance on these patients is still to be determined (Verduci et al., 2020; Mancilla et al., 2021). By modeling the microbiome-gut-brain axis (MGBA), it could be possible to trace single parameters that lead to specific alterations in the brain, which is challenging with current animal models (Raimondi et al., 2020). There is still a long way to go for establishing an entire (and sustainable) MGBA model, but advances are being made. Future MGBA models could open the door to important advancements not only in PKU, but also in a whole spectrum of diseases (Raimondi et al., 2020; CORDIS EU Research Results, 2021).

## Future Perspectves and Conclusion

PKU is a monogenic disease that leads to severe intellectual disability when untreated, and psychological sequelae in early-treated patients. Given the chronic nature of this disease, the complexity of the neuronal pathways affected and the possible relationship with neurodegenerative processes, it is of utmost importance to further clarify which mechanisms are involved, and how they are affected by high phenylalanine brain levels. Despite the relevance of the acquired knowledge, most currently available model systems have limited ability to recapitulate key aspects of PKU pathological processes. The 3D organoid systems that we describe in this review (Figure 2) represent a promising platform to further understand the neuropathology of PKU by providing tools to specifically address PKU-related targets and modulate key aspects of oxidative stress, brain L-Phe clearance, neurotransmitter deficiencies and LNAAs brain uptake. Furthermore, effects on the integrity of interneuron connectivity can also be further studied by fusing specific brain regions-derived organoids, known to be specially affected in PKU patients.

As mentioned, there is a myriad of unresolved issues regarding the complex processes that lead to brain damage in PKU. The convergence of multiple systems that combine different cell populations and different brain regions represent an exciting hope for resolving these questions. For now, most protocols showcase fetal developmental stages, which might be sufficient to address some neurodevelopmental related questions. The lack of maturation and vascularization represents a limitation in studying the relationship between PKU as a chronic disease, late-onset neurological symptoms and early aging brain. When this hurdle is overcome, such platforms will unlock our understanding of fundamental issues associated with age-related neurodegenerative processes associated to PKU. Without overlooking the limitations inherent to the current available organoid technology, these models could also pave the way for advanced pharmacological screening and pharmacological assessment, creating new opportunities for personalized medicine and prognosis prediction. Organoids could be seen as first in line to predict responsiveness and/or adverse reactions of specific groups of patients to available therapies and to test efficacy and safety of new therapeutic options. Additionally, as PKU shares neuronal symptomatology with other neurodegenerative disorders, understanding how brain processes are impacted by PKU might also provide a starting point for future studies on neurodegeneration.

## Author Contributions

AB and TF co-wrote the manuscript. All authors read and approved the final manuscript and contributed to the design, layout, and contents of the review.

## Conflict of Interest

The authors declare that the research was conducted in the absence of any commercial or financial relationships that could be construed as a potential conflict of interest.

## Publisher’s Note

All claims expressed in this article are solely those of the authors and do not necessarily represent those of their affiliated organizations, or those of the publisher, the editors and the reviewers. Any product that may be evaluated in this article, or claim that may be made by its manufacturer, is not guaranteed or endorsed by the publisher.
